# Transcriptomic and Biochemical Analysis Reveal Integrative Pathways Between Carbon and Nitrogen Metabolism in *Guzmania monostachia* (Bromeliaceae) Under Drought

**DOI:** 10.3389/fpls.2021.715289

**Published:** 2021-10-08

**Authors:** Ana Zangirolame Gonçalves, Helenice Mercier

**Affiliations:** Departamento de Botânica, Instituto de Biociências, Universidade de São Paulo (USP), São Paulo, Brazil

**Keywords:** CAM, ICDH, malate, N nutrition, PEPC, PEPCK, PPCK, urea

## Abstract

Most epiphytes are found in low-nutrient environments with an intermittent water supply. To deal with water limitation, many bromeliads perform crassulacean acid metabolism (CAM), such as *Guzmania monostachia*, which shifts from C_3_ to CAM and can recycle CO_2_ from the respiration while stomata remain closed during daytime and nighttime (CAM-idling mode). Since the absorbing leaf trichomes can be in contact with organic (urea) and inorganic nutrients (NO_3_^−^, NH_4_^+^) and the urea hydrolysis releases NH_4_^+^ and CO_2_, we hypothesized that urea can integrate the N and C metabolism during periods of severe drought. Under this condition, NH_4_^+^ can be assimilated into amino acids through glutamine synthetase (GS), while the CO_2_ can be pre-fixated by phosphoenolpyruvate carboxylase (PEPC). In this context, we evaluated the foliar transcriptome of *G. monostachia* to compare the relative gene expression of some genes involved with CAM and the N metabolism when bromeliads were submitted to 7days of drought. We also conducted a controlled experiment with an extended water deficit period (21days) in which bromeliads were cultivated in different N sources (urea, NH_4_^+^, and NO_3_^−^). Our transcriptome results demonstrated an increment in the expression of genes related to CAM, particularly those involved in the carboxylation metabolism (*PEPC1*, *PPCK*, and *NAD-MDH*), the movement of malate through vacuolar membrane (*ALMT9*), and the decarboxylation process (*PEPCK*). Urea stimulated the expression of *PEPC1* and *ALMT9*, while *Urease* transcripts increased under water deficit. Under this same condition, *GS1* gene expression increased, indicating that the NH_4_^+^ from urea hydrolysis can be assimilated in the cytosol. We suggest that the link between C and N metabolism occurred through the supply of carbon skeleton (2-oxoglutarate, 2-OG) by the cytosolic isocitrate dehydrogenase since the number of *NADP*-*ICDH* transcripts was also higher under drought conditions. These findings indicate that while urea hydrolysis provides NH_4_^+^ that can be consumed by glutamine synthetase-cytosolic/glutamate synthase (GS1/GOGAT) cycle, the CO_2_ can be used by CAM, maintaining photosynthetic efficiency even when most stomata remain closed 24h (CAM-idling) as in the case of a severe water deficit condition. Thus, we suggest that urea could be used by *G. monostachia* as a strategy to increase its survival under drought, integrating N and C metabolism.

## Introduction

Most epiphytic bromeliads inhabit oligotrophic environments with intermittent water and nutrient availability (Benzing, 2008). Periods of severe drought are frequent in the canopy, and it is important to understand how each species copes with harsh environments. One of the principal morphologic/anatomic features of bromeliads to face water and nitrogen (N) scarcity is the formation of a tank by the arrangement of leaves, such as a rosette; this structure is capable of accumulating water and dissolved nutrients, including organic N source, such as urea provided by the excrement of animals (Benzing and Bennett, 2000; Cambuí and Mercier, 2006). Highly efficient absorbing trichomes are distributed along the leaf blade following a gradient in which the basal part has the largest density and also the highest degree of cellular structure specialization (Kleingesinds et al., 2018). Alternatively, the apex has fewer trichomes and a greater number of stomata, thus playing an important role to take up CO_2_ from the atmosphere. A relevant adaptative physiological characteristic of many epiphytic tank bromeliads to face water stress is the crassulacean acid metabolism (CAM) photosynthesis because this process confers a higher carboxylation efficiency (Luttge, 2004) and a greater Rubisco (Ribulose-1,5-Biphosphate Carboxylase-Oxygenase) efficiency (Matiz et al., 2013; Gonçalves et al., 2020a).

To deal with nutrient scarcity of the canopy, the leaves of epiphytic tank bromeliads acquired the function of the roots of terrestrial plants. The leaf became the principal vegetative organ responsible for plant survival (Zotz, 2013), showing a spatial and functional division due to its ability to uptake nutrients, mainly in the basal region that is in contact with the tank solution, and to perform photosynthesis, mainly in the apical portion (Freschi et al., 2010; Takahashi and Mercier, 2011). Highly efficient uptake and great storage capacity of nutrients is another important physiological feature of the epiphytic tank bromeliads (Zotz and Asshoff, 2010). Effective leaf N uptake has been demonstrated for NH_4_^+^ and urea, which are the preferable N sources for *Vriesea gigantea* and *Guzmania monostachia* (Inselsbacher et al., 2007; Gonçalves et al., 2020b). Urea, after its intracellular hydrolysis by urease enzyme, provides NH_4_^+^ and CO_2_ to the leaf tissues (Miflin and Lea, 1980). Low and high affinity N transporters have been detected along the leaf blade of *G. monostachia*. At the base, for example, a significant increase of gene expression of NH_4_^+^ (*AMT1.2*) and urea (*DUR3* and *PIP1.2*) transporters were measured (Gonçalves et al., 2020b). Considering N assimilation, the highest activity of glutamine synthetase (GS) was found at the apex, while urease can be found at the base, indicating the importance of the apical foliar portion for amino acid production (Gonçalves et al., 2020b). Transcriptional analysis of *G. monostachia* leaf showed that the apex and middle portion had differentially expressed genes related to amino acid metabolism and photosynthesis, while the base showed responses to nutrients, particularly N (Mercier et al., 2019).

In its natural environment, *G. monostachia* can survive several months with the empty tank (Maxwell et al., 1995). To deal with water limitation, many bromeliad tanks exhibit CAM. This metabolism is a photosynthetic pathway that can be divided into four phases, in which CO_2_ is obtained overnight when stomata open (phase I) and it may be assimilated by the enzyme phosphoenolpyruvate carboxylase (PEPC), providing oxaloacetic acid (OAA) and subsequently forming malate by the cytosolic malate dehydrogenase (NAD-MDH; Luttge, 2004). Afterwards, malate is transported into the vacuoles through the aluminum-activated malate transporter (ALMT) family (Pereira et al., 2018) where it can be accumulated. At dawn (phase II), both PEPC and Rubisco are able to fix carbon while the stomata are still open (Matiz et al., 2013). In the subsequent light period (phase III), malic acid is remobilized to the cytosol and decarboxylated through NAD-MDH and phosphoenolpyruvate carboxykinase (PEPCK), producing CO_2_ that is consumed by Rubisco while stomata remain closed (Luttge, 2004). At dusk (phase IV), PEPC activity increases and Rubisco decreases while stomata reopen enabling CO_2_ assimilation (Luttge, 2004). In *G. monostachia*, for example, the isoform *ALMT9* is involved in the transport of both malate and fumarate to the vacuole (Pereira et al., 2018). During the subsequent light period, stomata remain closed while malic acid is remobilized from the vacuoles to the cytosol where it can be decarboxylated to phosphoenolpyruvate (PEP) and CO_2_ which is used for refixation in the C_3_ cycle (Luttge, 2004). *Guzmania monostachia*, as a facultative CAM species, can shift from C_3_ to CAM under drought and absorbs CO_2_ in the dark period (Medina et al., 1977). There is a variant of normal full CAM called CAM-idling in which stomata remain closed during night and day when water availability is unfavorable, like in a dry season. In this mode of CAM, respiratory CO_2_ is refixed in the dark period and recycled to carbohydrate during the light period (Lüttge, 2006). *Guzmania monostachia* showed a capacity to express a reversible CAM-idling photosynthesis under drought (Freschi et al., 2010), and it was estimated that the accumulation of nocturnal acid malic can contribute to approximately 50% of the total carbon assimilated under water deficit (Pikart et al., 2018). During drought, atmospheric CO_2_ absorption by the leaves is restricted due to stomata closure, causing a significant decrease in CO_2_ availability. Interestingly, the delivery of ^13^C-labeled urea to the leaves under water deficit enriched the ^13^C-malate pool of the apical portion of *V. gigantea* leaves (Matiz et al., 2017), suggesting that this organic N source could be an important source of CO_2_ for bromeliads under water stress (Matiz et al., 2017).

In this context, our goal was to investigate the connection between the N and C metabolism through urea nutrition during drought periods considering the *G. monostachia* bromeliad as a plant-model. Since urea hydrolysis releases CO_2_ and NH_4_^+^, we hypothesized that the interaction between drought and urea nutrition can activate the CAM pathway through the carboxylation metabolism (*PEPC1*, *PPCK*, and *NAD-MDH*), the movement of malate through vacuolar membrane (*ALMT9*), and the decarboxylation process (*PEPCK*), while NH_4_^+^ can be assimilated into glutamine through the activation of the GS/GOGAT cycle. Ultimately, the cytosolic isocitrate desidrogenase (NADP-ICDH) can participate in this process, providing 2-oxoglutarate (2-OG) from isocitrate. This 2-OG is then imported into the chloroplasts, where it can be used as the carbon skeleton required for amino acid synthesis.

## Materials and Methods

### Organism and Experimental Design

#### Gene Expression Related to CAM and N Metabolism

This experiment was conducted with adult *G. monostachia* (about 2.5years) obtained previously by *in vitro* propagation as described in Rodrigues et al. (2016). Until bromeliads reached this mature-vegetative age, they were kept in a greenhouse of the Department of Botany at the University of São Paulo (São Paulo, Brazil), where they received tap water daily and nutritive solution modified with half of the original concentration of macronutrients (Knudson, 1946) and with micronutrients (Murashige and Skoog, 1962) each, for 15days. These plants were kept in pots (14cm in diameter, 11cm high) containing a mixture of *Pinus* sp. bark and commercial organic substrate (Tropstrato®). These mature-vegetative bromeliads had around 20 leaves each and the oldest leaves measuring around 20cm length.

Previously to the experiment, bromeliads had their tanks emptied and were transferred to the growth chamber where they were kept for 1month under the following controlled conditions: 12-h photoperiod, photosynthetic photon flux of 250μmolm^−2^s^−1^, air temperature of 27/22°C day/night, and relative humidity of 60/70% day/night. During this period, all plants received distilled water daily and were not fertilized. After 30days, bromeliads were divided into two treatments (*n*=3 replicates): (1) bromeliads were subjected to 7days of water deficit (WD), and (2) bromeliads received distilled water for 7days (W). While the water treatment received distilled water for 7days, bromeliads of the water deficit treatment remained with the tank emptied throughout this period. Subsequently, apical and basal leaf portions of the 8th to 12th innermost nodes were harvested at 7am and 7pm, immediately frozen with liquid nitrogen, and stored at −80°C for RNA extraction.

#### CAM Modulation Under Different N Sources

In order to evaluate the interaction between CAM and nitrogen metabolism, the experiment was carried out with adult bromeliads in the same controlled conditions of temperature, light, relative humidity, and photoperiod as described above. After 30days, bromeliads received nutrient solutions with a total of 5mM of nitrogen, according to the following treatments (*n*=8 replicates per treatment): (1) without nitrogen source (control), (2) Ca(NO_3_)_2_ (nitrate), (3) (NH_4_)_2_SO_4_ (ammonium), and (4) urea (urea). The nutrient solution was prepared with half of the original concentration of macronutrients (Knudson, 1946; modified without nitrogen sources) and with micronutrients (Murashige and Skoog, 1962). Each experimental bromeliad received 30ml of the nutrient solution inside its tank once every 7days for 30days (totaling four applications).

Twenty-four hours after the last nutrient-solution application, bromeliads had their tanks emptied and were separated into two new treatments for CAM induction (*n*=4 replicates per treatment): (1) receiving distilled water to keep the tank full of water (W) and (2) water deficit condition, in which bromeliads did not receive distilled water (WD). Bromeliads remained in these conditions for 21days as this environmental condition was shown to be the best to induce the transient shift of the photosynthesis from C_3_ to CAM-idling in the apical part of the leaves of *G. monostachia* (Pikart et al., 2020). At the end of the experiment, the apical leaf portion of the 8th to 12th innermost nodes were harvested at 7am in order to analyze the relative water content (RWC) in the leaves (RWC), the activity of the enzyme PEPC, the expression of the CAM-specific phosphoenolpyruvate carboxylase gene (*PEPC1*), and the tonoplast aluminum-activated malate transporter gene (*ALMT9*). For the quantification of nocturnal acid accumulation (ΔH^+^), bromeliad leaves were collected at 7am and 7pm.

### RNA Extraction, RNA-Seq *de novo* Assembly, Annotation, and Analysis

Total RNA was extracted using the PureLink® RNA Mini Kit (Ambion) according to the manufacturer’s protocol. RNA concentration, quality, and integrity were checked as described in Mercier et al. (2019). Samples with integrity number ≥6.5 were pooled to generate libraries using TruSeq RNA Sample Preparation Kit, Set A (Illumina Inc., United States). Paired-end sequences were generated as described by Mercier et al. (2019), and only high-quality sequences (with average PhredScore over 24) were used. Sequence normalization and *de novo* transcriptome assembly were performed using Trinity (Grabherr et al., 2011; Haas et al., 2013) as described in Mercier et al. (2019), and the reads were deposited at NCBI at the Bioproject ID PRJNA532595. *Guzmania monostachia* transcripts were annotated using the Viridiplantae (taxa ID 33090, VP) and Monototyledons (taxa ID 4447, MC) database through the BLAST suite (Camacho et al., 2008) and Blast2GO^1^ (Conesa et al., 2005). The annotated genes related to *NR* (nitrate reductase), *NiR* (nitrite reductase), *Urease* (urease), *GS2* (glutamine synthetase-chloroplastic), *GS1* (glutamine synthetase-cytosolic), *Fd-GOGAT* (ferredoxin-glutamate synthase), *NADH-GOGAT* (NADH-glutamate synthase), *NADP-ICDH* (NADP-isocitrate dehydrogenase), *PEPC1* (phosphoenolpyruvate carboxylase), *PPCK1* (phosphoenolpyruvate carboxylase kinase), *NAD-MDH* (NAD-malate dehydrogenase), *ALMT9* (tonoplast aluminum-activated malate transporter), and *PEPCK* were selected and analyzed through pairwise differential expression (DE) as described by Mercier et al. (2019), and the heatmap was generated using the Morpheus tool^2^ in order to compare apex and base, samples collected at 7am and 7pm, and water (W) and water deficit (WD) treatments.

### Relative Water Content

The RWC of the plants was determined by collecting 10 leaf discs of a known area, from which the values of fresh mass (MF), and, subsequently, the discs remained in distilled water for 24h to obtain the turgid mass (MT). After this period, the discs were dried at 60°C for 48h to determine the dry mass (DM). These results were used in the following equation: RWC (%)=[(MF-MS)/(MT-MS)]*100 (Weatherley, 1950).

### Phosphoenolpyruvate Carboxylase Enzyme Activity

The determination of PEPC activity was performed by the *in vitro* method as described by Nievola et al. (2001) and adapted for *G. monostachia* (Freschi et al., 2010; Pereira et al., 2013). In this determination, samples of the apical portion of leaves (1g) were collected, macerated in liquid nitrogen and immersed in 5ml of the extraction buffer (pH 8), composed of 200mM Tris–HCl, 10mM MgCl_2_, 5mM DTT, 1mM EDTA, 0.5% (w/v) BSA, and 10% (v/v) glycerol. The samples were centrifuged for 2min at 15,000*g*. The supernatant was collected and passed through a SEPHADEX PD10/G-25 column. PEPC activity was analyzed in 2ml of reaction buffer containing 50mM Tris–HCl (pH 8.0), 1mM DTT, 10mM MgCl_2_, 100mM NaHCO_3_, 20mM NADH, and 3mM phosphoenolpyruvate (PEP). The reaction was started by adding 200μl of the extract. The consumption of NADH was quantified in a spectrophotometer (340nm) at the initial moment of the reaction and after 4min. The results were expressed in micromoles of NADH consumed per minute per gram of dry mass (μmolmin^−1^g^−1^).

### Nocturnal Acid Accumulation

The apical leaf portion was analyzed for the degree of CAM expression according to the nocturnal acid accumulation (ΔH^+^) using the titratable acidity methodology (Matiz et al., 2017). For this, the leaves of the bromeliads were cut into small fragments of 1cm^2^, and 0.1g was macerated in liquid nitrogen, homogenized in 500μl of MCW solution, and incubated at 60°C for 30min. The MCW solution was prepared in a proportion of 12:5:1 of methanol:chloroform:water, respectively. Subsequently, the tubes received 500μl of distilled water and were centrifuged for 10min at 16,000*g*. After centrifugation, 1.5ml of the supernatant was collected and completely dried at 60°C. After this procedure, the supernatant was collected and stored at −20°C until the titratable acidity procedure.

For the titratable acidity procedure, a solution of phenolphthalein (10mg in 10ml of ethanol) was diluted in 3ml of boiled distilled water to be pipetted into an Elisa® plate. In the microplate, the following volumes per sample were pipetted in this order: 100μl of boiled distilled water, 100μl of phenolphthalein, 5μl of the respective sample, and 100μl of the curve solution. All samples received all points on the curve in order to calculate their H^+^ concentrations.

Curve points were obtained as follows: (A) first point with 0.5μmol of H^+^ (250μl of 0.2M NaOH and 9.75ml of boiled distilled water), (B) second point with 0.4μmol of H^+^ (200μl of 0.2M NaOH and 9.80ml of boiled distilled water), (C) third point with 0.3μmol of H^+^ (150μl of 0.2M NaOH and 9.85ml of boiled distilled water), (D) fourth point with 0.2μmol of H^+^ (100μl of 0.2M NaOH and 9.90ml of boiled distilled water), (E) fifth point with 0.16μmol of H^+^ (80μl of 0.2M NaOH and 9.92ml of boiled distilled water), (F) sixth point with 0.12μmol of H^+^ (60μl of 0.2M NaOH and 9.94ml of boiled distilled water), (G) seventh point with 0.08μmol of H^+^ (40μl of 0.2M NaOH and 9.96ml of boiled distilled water), and (H) eighth point with 0.04μmol of H^+^ (20μl of 0.2M NaOH and 9.98ml of boiled distilled water).

### Gene Expression

The genes encoding the expression of the CAM-specific PEPC enzyme (*PEPC1*) and the tonoplast aluminum-activated malate transporter (*ALMT9*) which presented the most discrepant gene expression in the transcriptome of *G. monostachia* (Mercier et al., 2019) were selected for the primer design using the PerlPrimer software^3^ (Supplementary Table S2). Among all *PEPC* isoforms found in the transcriptome, *PEPC1* was selected because its genomic sequence was more similar to the genomic sequences of the CAM-specific PEPC enzyme comparing to other CAM species (Beltran and Smith, unpublished data). In addition, *ALMT9* isoform was selected because it showed to be involved in malate transport to the vacuole in *G. monostachia* (Pereira et al., 2018). The reference genes (beta-amylase, *BAM*; betahydroxysteriod-dehydrogenase decarboxylase, *HSDD*; Supplementary Table S2) were selected from the *G. monostachia* transcriptome as described by Gonçalves et al. (2020b).

Total RNA of bromeliad leaves was extracted from 300mg using Trizol® reagent (Invitrogen®) with the PureLink® RNA mini kit (Life Technologies®), according to the manufacturer instructions. RNA concentration, its integrity analysis, and the removal of DNA contamination have been described previously by Gonçalves et al. (2020b). The cDNA was synthesized using the SuperScript IV (Invitrogen®) reaching a final volume of 20μl according to the manufacturer instructions. The PCRs were performed using the StepOnePlusTM® Real-Time PCR (Applied Biosystems®), and products were detected using SYBR® Green PCR Master Mix (Applied Biosystems®). The analyses of gene expressions were performed using BestKeeper (Pfaffl et al., 2004).

### Statistical Analyses

The response variables of the RWC, the activity of the PEPC enzyme, the nocturnal acid accumulation (ΔH^+^), and the expression of the genes *PEPC1* and *ALMT9* were compared using ANOVA, and Tukey HSD *post hoc* tests were used for pair-wise comparisons. All statistical analyses were performed with the statistical platform R (R Core Team, 2020).

## Results

### Gene Expression Related to CAM

**Figure 1:**
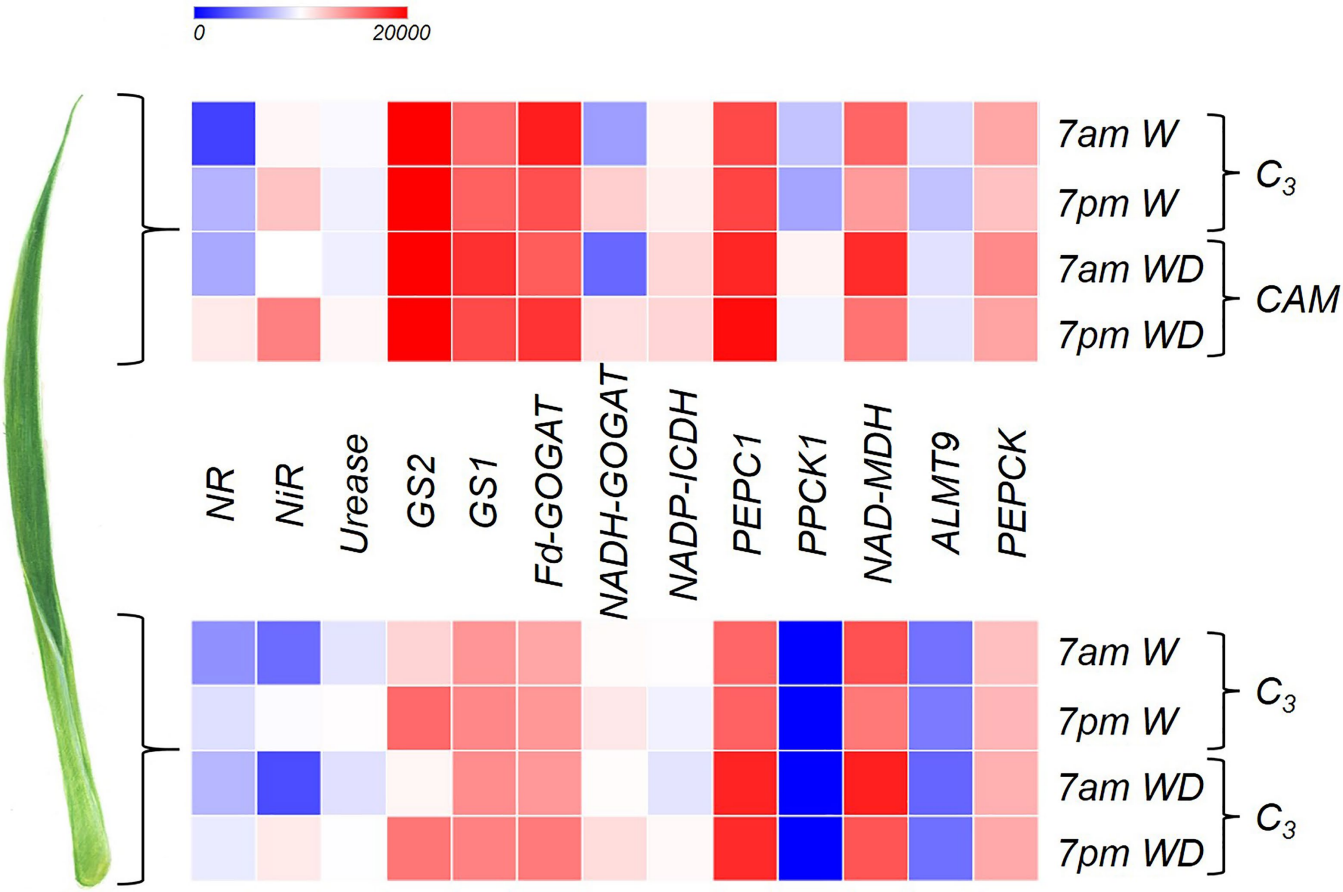
Heatmap indicating log_2_ (fold-change) of the genes *NR* (nitrate reductase), *NiR* (nitrite reductase), *Urease* (urease), *GS2* (glutamine synthetase-chloroplastic), *GS1* (glutamine synthetase-cytosolic), *Fd-GOGAT* (Ferredoxin- glutamine-2-oxoglutarate aminotransferase), *NADH-GOGAT* (NADH- glutamine-2-oxoglutarate aminotransferase), *NADP-ICDH* (NADP-isocitrate dehydrogenase), *PEPC1* (phosphoenolpyruvate carboxylase), *PPCK1* (phosphoenolpyruvate carboxylase kinase), *NAD-MDH* (NAD-malate dehydrogenase), *ALMT9* (tonoplast aluminum-activated malate transporter), and phosphoenolpyruvate carboxykinase (*PEPCK*) in order to compare the apical and basal leaf portions of *Guzmania monostachia* submitted to 7days of water deficit (WD) or receiving distilled water (W) for the same period. Samples were collected at 7am and 7pm in order to compare gene expressions in different periods of the day. More details about the methodology are described in the section “Gene Expression Related to CAM and N Metabolism.” Heatmap colors varied from blue to red representing low to high gene expression.

Some genes related to CAM showed markedly greater expression in the leaf apex of plants subjected to WD, especially *PPCK1* and *ALMT9* (Figure 1) when compared to the base. *PEPC1* showed a high gene expression throughout the entire leaf; however, its highest expression was observed at 7am under WD (Figure 1). *NAD-MDH* showed greater number of transcripts at 7am independent of the leaf portion, especially when the plant was subjected to WD (Figure 1). The highest expression of *PEPCK* was observed at the leaf apex at 7am under WD (Figure 1).

### Gene Expression Related to N Metabolism

Both *NR* and *NiR* had the highest relative gene expression at 7pm in the apex of the bromeliad leaves subjected to WD (Figure 1). Similarly, the quantity of urease transcripts in the apical leaf portion were the highest at 7pm under drought (Figure 1). *GS2* showed greater gene expression at the leaf apex compared to the base, independent of the treatments and time of harvest; however, for the base the *GS2* expression was stronger at 7pm, independent of the water availability (Figure 1). Similar to *GS2*, *GS1* showed higher gene expression at the leaf apex, especially at 7am, when plants were subjected to WD (Figure 1). The gene expression of *Fd-GOGAT* was greater at the leaf apex compared with the base, while at the basal part its expression at 7pm under WD was more intense compared with the other treatments (Figure 1). The *NADH-GOGAT* showed higher gene expression at 7pm with the apex showing a greater quantity of transcripts than the basal part (Figure 1). The transcripts of *NADP*-*ICDH* were more abundant in the apical leaf portion compared to the base, mainly under water deficiency.

### CAM Modulation Under Different N Sources

**Figure 2:**
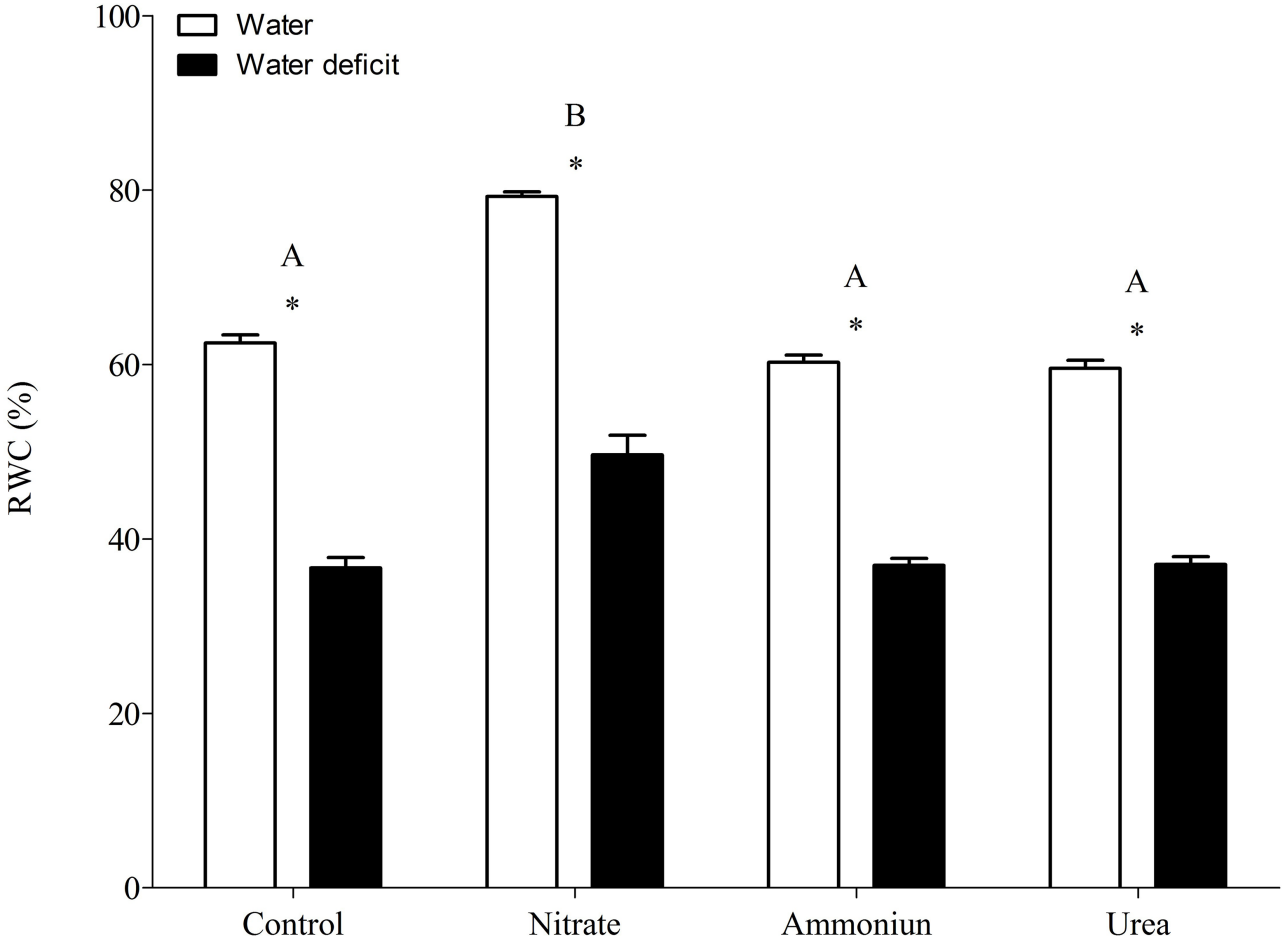
Relative water content in the apical leaf portion of *G. monostachia* submitted to the following treatments for 1 month: nutrient solution without nitrogen source (control), Ca(NO_3_)_2_ (nitrate), (NH_4_)_2_SO_4_ (ammonium), and urea (urea). Twenty-four hours after the last application of the nutrient solutions, bromeliads were separated into two new treatments for 21days: (1) receiving distilled water (water) and (2) water deficit condition in which the bromeliads did not receive distilled water (water deficit). Bars indicate standard error. Letters indicate statistical differences between nutritional treatments, and asterisk (*) indicates differences between water vs. water deficit (ANOVA/Tukey HSD *post hoc* test, *α*=0.05). Letters are showing the statistical differences among nutritional treatments also under water treatments. Since the nutritional vs. water treatment was statistically significant (Supplementary Table S1), we represented each nutritional treatment with a single letter.

**Figure 3:**
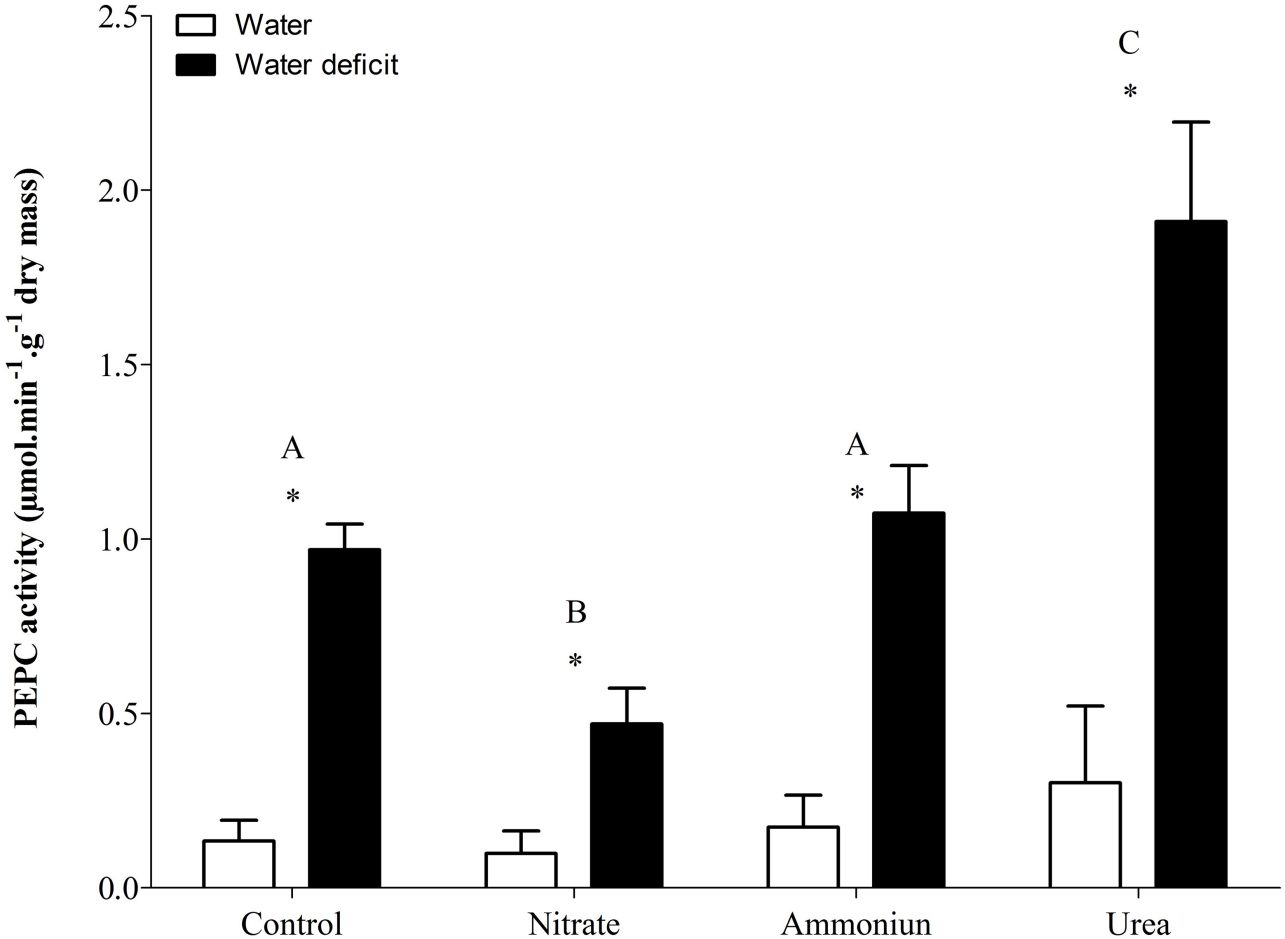
Activity of the phosphoenolpyruvate carboxylase (PEPC) enzyme in the apical leaf portion of *G. monostachia* submitted to the following treatments for 1month: nutrient solution without nitrogen source (control), Ca(NO_3_)_2_ (nitrate), (NH_4_)_2_SO_4_ (ammonium), and urea (urea). Twenty-four hours after the last application of the nutrient solutions, bromeliads were separated into two new treatments for 21days: (1) receiving distilled water (water) and (2) water deficit condition in which the bromeliads did not receive distilled water (water deficit). Bars indicate standard error. Letters indicate statistical differences between nutritional treatments, and asterisk (*) indicates differences between water vs. water deficit (ANOVA/Tukey HSD *post hoc* test, *α*=0.05). Letters are showing the statistical differences among nutritional treatments also under water treatments. Since the nutritional vs. water treatment was statistically significant (Supplementary Table S1), we represented each nutritional treatment with a single letter.

**Figure 4:**
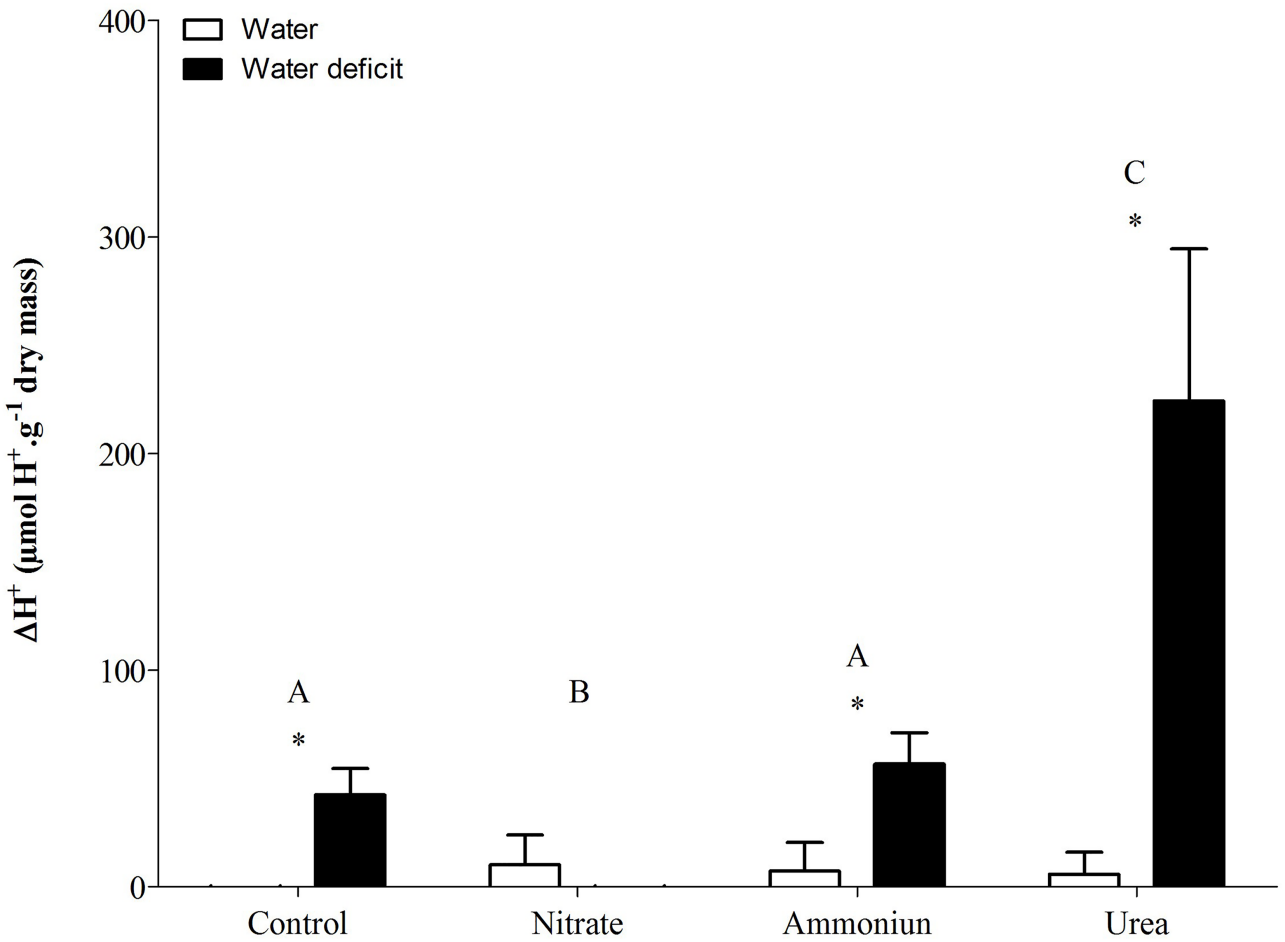
Nocturnal acid accumulation (ΔH^+^) in the apical leaf portion of *G. monostachia* submitted to the following treatments for 1month: nutrient solution without nitrogen source (control), Ca(NO_3_)_2_ (nitrate), (NH_4_)_2_SO_4_ (ammonium), and urea (urea). Twenty-four hours after the last application of the nutrient solutions, bromeliads were separated into two new treatments for 21days: (1) receiving distilled water (water) and (2) water deficit condition in which the bromeliads did not receive distilled water (water deficit). Bars indicate standard error. Letters indicate statistical differences between nutritional treatments, and asterisk (*) indicates differences between water vs. water deficit (ANOVA/Tukey HSD *post hoc* test, *α*=0.05). Letters indicate statistical differences between nutritional treatments, and asterisk (*) indicates differences between water vs. water deficit (ANOVA/Tukey HSD *post hoc* test, *α*=0.05). Letters are showing the statistical differences among nutritional treatments also under water treatments. Since the nutritional vs. water treatment was statistically significant (Supplementary Table S1), we represented each nutritional treatment with a single letter.

Bromeliads that were subjected to WD and received urea, ammonium or did not receive N showed the lowest RWC in the leaves, while the plants that were fertilized with nitrate had the highest water content (Supplementary Table S1; Figure 2). Hydrated plants that were fertilized with nitrate showed the highest RWC and, after 21days of WD, those bromeliads kept 50% of their water content (Supplementary Table S1; Figure 2). Consistent with these results, we observed that the PEPC enzyme activity and the nocturnal acid accumulation were higher in bromeliads that acquired urea and were submitted to WD, while bromeliads that took up nitrate with WD showed lower PEPC activity and nocturnal acid accumulation (Supplementary Table S1; Figures 3, 4). In addition, bromeliads that received ammonium or did not receive N at all and were subjected to WD presented intermediate PEPC activity and nocturnal acid accumulation (Supplementary Table S1; Figures 3, 4). On the other hand, bromeliads showed the lowest PEPC activities and nocturnal acid accumulation when they received water regardless of nutritional treatment (Supplementary Table S1; Figures 3, 4).

**Figure 5:**
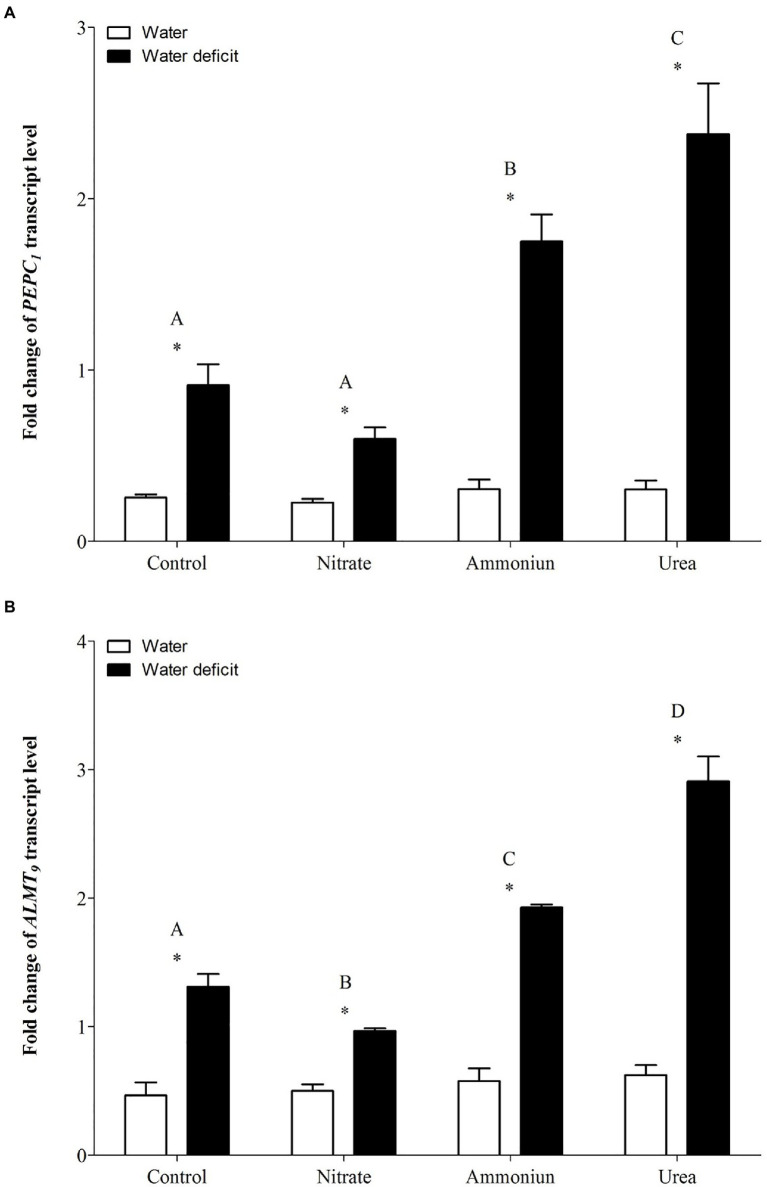
Gene expression (*fold change*) of the **(A)** phosphoenolpyruvate carboxylase gene (*PEPC1*) and **(B)** the tonoplast aluminum-activated malate transporter gene (*ALMT9*) in the apical leaf portion of *G. monostachia* submitted to the following treatments for 1month: nutrient solution without nitrogen source (control), Ca(NO_3_)_2_ (nitrate), (NH_4_)_2_SO_4_ (ammonium), and urea (urea). Twenty-four hours after the last application of the nutrient solutions, bromeliads were separated into two new treatments for 21days: (1) receiving distilled water (water) and (2) water deficit condition in which the bromeliads did not receive distilled water (water deficit). Bars indicate standard error. Letters indicate statistical differences between nutritional treatments, and asterisk (*) indicates differences between water vs. water deficit (ANOVA/Tukey HSD *post hoc* test, *α*=0.05). Letters indicate statistical differences between nutritional treatments, and asterisk (*) indicates differences between water vs. water deficit (ANOVA/Tukey HSD *post hoc* test, *α*=0.05). Letters are showing the statistical differences among nutritional treatments also under water treatments. Since the nutritional vs. water treatment was statistically significant (Supplementary Table S1), we represented each nutritional treatment with a single letter.

Concerning the gene expression of *PEPC1*, a significant increase in its expression was observed in bromeliads that acquired urea under WD, followed by plants that were fertilized with ammonium under WD (Supplementary Table S1; Figure 5A). On the other hand, plants that received nitrate or did not receive N and were subjected to WD showed lower *PEPC1* gene expressions comparing to bromeliads with other nutritional treatments (Figure 5A). Additionally, hydrated plants presented the lowest *PEPC1* gene expressions regardless the nitrogen they received (Supplementary Table S1; Figure 5A). Consistently, *ALMT9* showed its highest expression in bromeliads that acquired urea and were submitted to WD, and the lowest expression was observed in plants that were nourished with nitrate followed by WD (Supplementary Table S1; Figure 5B). Bromeliads that received ammonium or did not have contact with any N sources and were submitted to WD showed intermediate *ALMT9* gene expressions (Supplementary Table S1; Figure 5B). Likewise, the gene expressions of *ALMT9* were lower in hydrated plants comparing to WD plants (Supplementary Table S1; Figure 5B).

## Discussion

**Figure 6:**
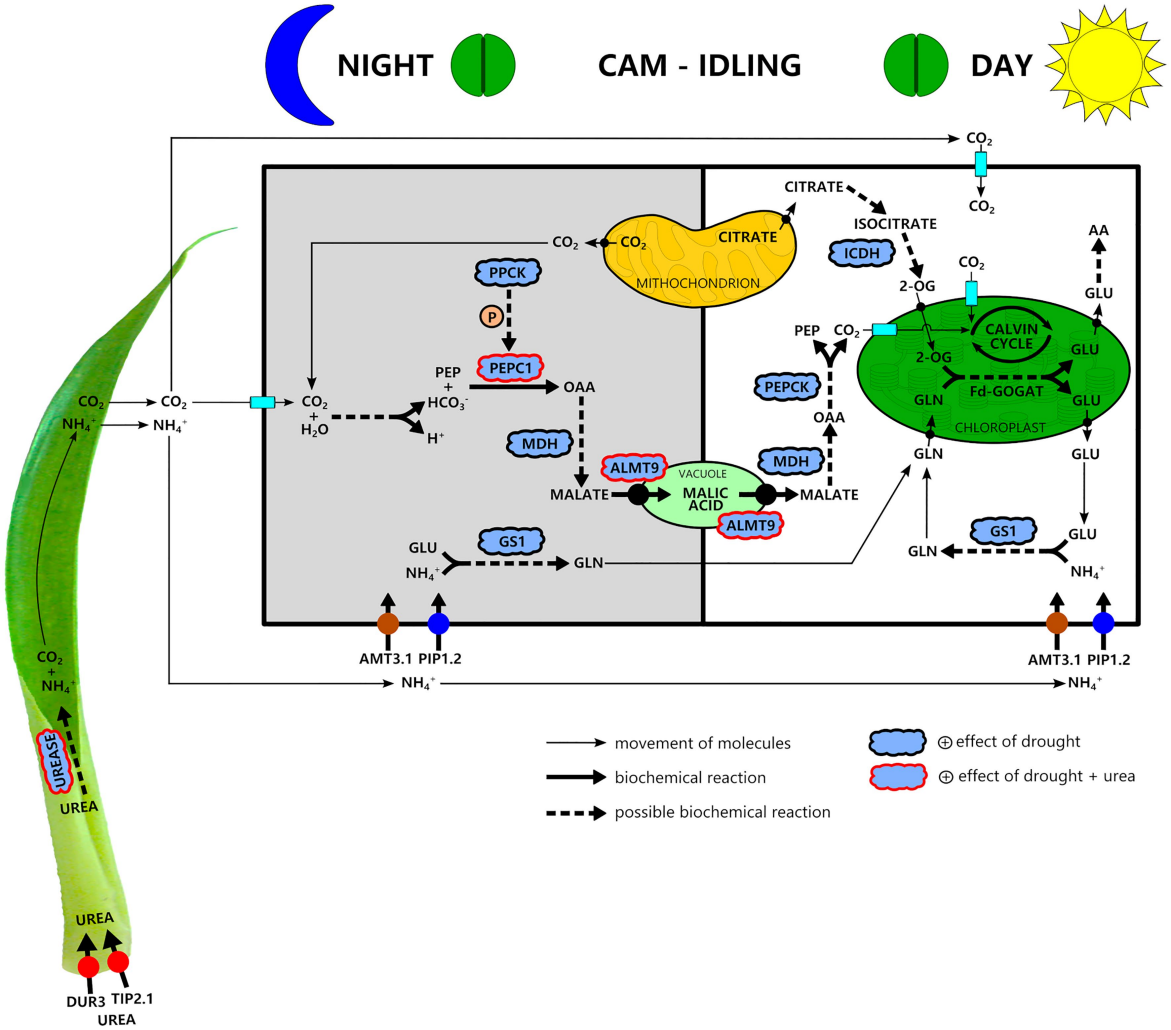
A simplified schematic showing our hypothesis about nitrogen metabolism and its interaction with carbon metabolism in a water stress-induced CAM-idling bromeliad, *G. monostachia*, submitted to urea nutrition. Urea is taken up at the leaf basal part and after its hydrolysis generates NH_4_^+^ and CO_2_. At the green leaf part and mainly in the apex portion during the night, the CO_2_ may be assimilated through the crassulacean acid metabolism (CAM) pathway. Urea nutrition associated with water stress positively stimulated *PEPC1* and *ALMT9* gene expressions and PEPC1 activity, resulting in a higher nocturnal vacuolar acidity. NH_4_^+^, in turn, mainly after entering a cell of the apical leaf region may be assimilated during the light–dark period in the cytosol mediated by the enzyme GS1 forming glutamine (Gln). Therefore, glutamine and glutamate (Glu) biosynthesis may occur in separate compartments. The mitochondria may supply the carbon skeleton for Fd-GOGAT functioning. The key C-compound, 2-oxoglutarate (2-OG), may derive from sugar respiration reactions. In this pathway, citrate is used to generate the isocitrate required for 2-OG synthesis, allowing the net Glu production in the chloroplast and then other amino acid formation. Abbreviations for enzymes: PEPC1, phosphoenolpyruvate carboxylase 1; PPCK, phosphoenolpyruvate carboxylase kinase; NAD-MDH, NAD-malate dehydrogenase; PEPCK, phosphoenolpyruvate carboxykinase; Rubisco, ribulose-1,5-bisphosphate carboxylase/oxygenase; GS1, glutamine synthetase 1; Fd-GOGAT, glutamine-2-oxoglutarate aminotransferase; and NADP-ICDH, NADP-isocitrate dehydrogenase. Abbreviations for transporters: ALMT9, tonoplast aluminum-activated malate transporter 9; DUR3, urea transporter; AMT3.1, ammonium transporter; TIP2.1, tonoplast intrinsic protein; PIP1.2, plasma membrane intrinsic protein; AA, amino acid.

Our results indicate that drought had a positive effect on the C_3_-CAM shift at the apical leaf part, increasing the expression of genes related to carboxylation metabolism (*PEPC1*, *PPCK*, and *NAD-MDH*), movement of malate through vacuolar membrane (*ALMT9*) and decarboxylation process (*PEPCK*; Figure 6). Together with water deficiency, urea hydrolysis seemed to provide CO_2_ for malate production and accumulation into vacuoles since we observed that urea stimulated the expression of *PEPC1* and *ALMT9* genes (Figure 6). At the same time, urea hydrolysis provides NH_4_^+^(Miflin and Lea, 1980) that can be assimilated into Gln since our results showed increased *GS1* gene expression under water deficiency (Figure 6). Ultimately, *NADP-ICDH* can supply the carbon skeleton to Fd-GOGAT functioning (Figure 6). Because *G. monostachia* is an epiphytic bromeliad subjected to the intermittence of water and nutrients in its natural environment, we reinforce that urea nutrition obtained in nature by the symbiotic interaction of bromeliads with anurans may bring advantages since the CO_2_ can maintain the photosynthetic efficiency even when most stomata remain 24h closed, while NH_4_^+^ can maintain amino acid and protein production.

### C3/CAM-Idling Comparison Through RNA-Seq

Our bromeliads were submitted to drought, and this environmental condition was shown to be the best to induce the transient shift of the photosynthesis from C_3_ to CAM-idling in the apical part of the leaves of *G. monostachia* (Freschi et al., 2010; Mercier et al., 2019). However, the molecular mechanisms underlying the stress induction of the transition from C_3_ to CAM remain poorly understood. Recently, this was observed for the facultative C_3_-CAM plant *Mesembryanthemum crystallinum* (Aizoaceae), whose guard cells themselves can shift from C_3_ to CAM, increasing expression of *PEPC1* and *PPCK1*, which encode key enzymes of CAM photosynthesis (Kong et al., 2020).

Comparison of the differential patterns of mRNA abundance in the apical leaf portion between C_3_ (W treatment) and CAM-idling (WD treatment) of *G. monostachia* tissues suggests that the C_3_-CAM switch may be determined by the greater expressions of some genes involved in the carboxylation metabolism (*PEPC1*, *PPCK*, and *NAD-MDH*), the movement of malate through vacuolar membrane (*ALMT9*) and the decarboxylation process (*PEPCK*), independent of harvest time (7am or 7pm). It is interesting to observe that in the basal portion, although there was an increase in the number of *PEPC1* transcripts in drought condition, there was practically no expression of *PPCK1*. This result indicates that the enzyme PEPC1 could not be intensely activated through the phosphorylation process, and, consequently, the CO_2_ fixation could not happen through the CAM pathway in this part of the leaf. Besides that, the basal part of the leaves has fewer stomata in relation to the middle and apex portions and this morphology may cause some degree of restriction of CO_2_ influx (Freschi et al., 2010). Previous studies have demonstrated that there is an upregulation of *PEPC1* expression (7am, 12pm, and 5pm) in the leaf apex of *G. monostachia* submitted to 20d of water deficit. However, it was interesting to note that there is also a diurnal modulation of decarboxylation activity of PEPCK with higher gene expression at 7am and an increase in the enzyme activity at noon, avoiding a futile cycling of organic acid during CAM (Pikart et al., 2020).

Considering that the plants were under nutritional restrictions and the base part was not in contact with N, the RNA-seq analysis showed a higher *NR* and *NiR* transcript accumulation following water deficit at 7pm in the foliar apex. In addition to the primary role in N uptake and assimilation, the enzyme nitrate reductase has also been described to possess a nitrite-NO reductase activity that uses nitrite to produce nitric oxide (NO; Astier et al., 2018). This signaling molecule could be involved with stomatal closure in response to plant acclimation to drought (Mioto et al., 2014). Detached leaves of *G. monostachia* exposed for 7days to polyethylene glycol showed that NO increased exclusively in the apical part of the leaves and the stomata remained closed even in the night period (CAM-idling mode; Mioto and Mercier, 2013). In *Sedum album*, another facultative C_3_-CAM plant (Crassulaceae), an increase in NR activity enzyme and a corresponding increase in endogenous NO levels were observed under drought treatment. This rise in NO level occurred during C_3_-CAM transition, correlating well with CAM expression (Habibi, 2020).

Photosynthesis and N metabolism are closely interconnected with the latter being a sink for ATP, reduced power and carbon skeletons produced during photosynthesis (Lea and Ireland, 1999). NH_4_^+^, whether converted from nitrate or generated by urea hydrolyzation, is assimilated *via* the GS/glutamine-2-oxoglutarate aminotransferase (GOGAT) cycle, which can be considered very important to maintain NH_4_^+^ at low concentrations in the leaves, helping to counteract ROS formation due to the consumption of reductants, such as Fdred and NADH (Lea and Miflin, 2003). In the context of nutritional deficiency, as the transcriptome of *G. monostachia* was performed, the base of the leaves presented markedly lower transcript levels of *GS2*, *GS1*, *Fd* or *NADH-GOGAT*, *NADP-ICDH* and *urease* genes. *GS1* transcripts increased in the apical part in response to water stress, suggesting some importance in the *G. monostachia* CAM-idling shift. McNally et al. (1983), studying some C_4_-type and CAM plants, observed that the foliar GS1 enzyme can contribute 35–80% of total GS-activity. Previous work with epiphytic tank bromeliads, such as *V. gigantea* and *G. monostachia*, demonstrated that assimilation of NH_4_^+^ took place mainly in the apical leaf part (Takahashi and Mercier, 2011; Gonçalves et al., 2020b). The enzyme GS1 is located in the cytosol and plays an important role in the primary NH_4_^+^ assimilation from the environment (Sakakibara et al., 1996). In pine and other conifers NH_4_^+^, which is the predominant source of N absorbed for these trees, is assimilated in the cytosol through GS1, and glutamine (Gln) and glutamate (Glu) biosynthesis occur in the chloroplast (Suárez et al., 2002). Tomato GS1/GOGAT cycle was related to stress responsiveness since their expression levels were modified under drought, cold or heat stress treatments (Liu et al., 2016). On the other hand, *GS2* and *Fd*- or *NADH-GOGAT* transcripts of *G. monostachia* did not show any increase under water deficiency, and *GS2*/*Fd-GOGAT* genes were highly expressed in the apical leaf part. It is well known that the chloroplastidic GS2/Fd-GOGAT cycle plays important roles in the assimilation of N from NO_3_^−^ in leaf and in plant survival under photorespiratory conditions which lead to the generation of excess NH_4_^+^ accumulation (Krapp, 2015), while NADH-GOGAT is crucial for NH_4_^+^ assimilation in heterotrophic tissues (Selinski and Scheibe, 2019). For *Mesembryanthemum crystalinum*, after the transition from C_3_ to CAM the plants still maintain the capacity to photorespire (Whitehouse et al., 1991). In a previous study using CAM-induced leaves of *G. monostachia*, it was observed that *glycolate oxidase 1* (*GLO1*) mRNA abundance, which is a gene considered a photorespiration indicator, presented higher expression at noon in CAM-induced plant compared to the dawn and dusk times (Pikart et al., 2020). The shift from C_3_ to CAM-idling in *G. monostachia* leaf apex could be related to the increase in the *NADP-ICDH* gene expression, which encodes the cytosolic enzyme isocitrate dehydrogenase. This enzyme allows a net synthesis of Glu, providing the key organic acid 2-oxoglutarate (2-OG), which is then transported into plastids allowing GOGAT functioning (Hodges, 2002). Since 2-OG could play a significant role modulating the flow of C to N metabolism depending upon environmental conditions (Foyer et al., 2011), it is possible that changes in its synthesis could reflect the decrease of internal CO_2_ levels caused by day/night stomatal closure, which is typical of the CAM-idling photosynthesis.

### Urea Can Positively Modulate C3-CAM Shift Under Water Deficit

*Guzmania monostachia*, as an example of epiphytic tank bromeliad, is a good model to study the effects of organic nitrogen nutrition on bromeliad development and, particularly, on photosynthetic plasticity when exposed to water deficit (Rodrigues et al., 2014, 2016). In the low-nutrient epiphytic environment, which frequently presents nitrogen and water scarcity, organic compounds such as urea could represent an important nutritional resource that provides both nitrogen and carbon (Matiz et al., 2017). Our investigation reveals for the first time that urea can positively modulate the C_3_-CAM shift when the leaves were under drought stress. Leaves from urea treatment showed the highest increases in the expressions of *PEPC1* and *ALMT9* genes comparing with inorganic N sources (NO_3_^−^ or NH_4_^+^) and, consequently, the greatest levels of PEPC activity and nocturnal acid accumulation. Therefore, it seems that the change from C_3_ to CAM mode was stimulated by this organic N source. On the other hand, plants supplied with NO_3_^−^ were less vulnerable to water loss under drought conditions while showed lower expressions of *PEPC1* and *ALMT9* genes. Nitrate is known to increase the endogenous cytokinin level, which may regulate negatively the PEPC activity and decrease CAM in *G. monostachia* leaves (Pereira et al., 2013, 2018). Furthermore, NO_3_^−^ uptake could be accompanied by K^+^ absorption in *Arabidopsis thaliana* as a charge balance, which can enable osmotic adjustments and may keep the RWC in this plant (Wang et al., 2012; Rubio et al., 2014). Therefore, the maintenance of water content may delay or down-regulate the CAM.

It is well known that abiotic stresses, such as drought, induce stomata closure and, consequently, limit uptake of atmospheric CO_2_ into the leaf tissue (Chen et al., 2018; Gobara et al., 2020). In this context, urea hydrolysis through foliar urease activity may partially compensate for the CO_2_ reduction by providing it, in addition to NH_4_^+^ (Matiz et al., 2017, 2019). This mechanism may represent an important physiological adaptation to obtain C and N at the same time. For *G. monostachia*, urea and NH_4_^+^ are the preferred N forms of uptake (Gonçalves et al., 2020b). The absorption of urea as a whole molecule took place mainly at the basal region of the leaf through the transporters of low (TIP2.1) and high (DUR 3) affinity and may be stored in the vacuole before the water deficit imposition (Matiz et al., 2019; Gonçalves et al., 2020b). If we consider the CAM-idling photosynthetic mode, whose gas exchange with the atmosphere largely ceases and respiration provides the CO_2_ (Winter, 2019), this strategy of using urea as a preferential source of N becomes even more relevant. Upper leaf parts of the C_3_ tank epiphytic bromeliad *V. gigantea* which close their stomata during the day under drought had a greater increase in urease activity after being supplied with urea at nighttime when compared to daytime. At the same time, a higher PEPC activity was found, indicating that at least part of the CO_2_ produced by urea hydrolyses was fixed during the night (Matiz et al., 2017). Furthermore, a cytochemical detection of CO_2_ was performed in the apex leaf portion after urea application during the night, and CO_2_ deposition was detected in a greater quantity in the cytoplasm near the chloroplasts than in well-watered leaves (Matiz et al., 2017).

It seems that urease activity occurs during both the day and night in *G. monostachia* leaves since we observed an increase in the expression of *urease* and *GS1* genes under water scarcity. Thus, we consider that the first step of NH_4_^+^ assimilation may take place in the cytosol of this bromeliad through GS1 enzyme, giving raise to glutamine (Gln) concentration that could enter the chloroplast. Subsequently, the enzyme GOGAT transfers the amide group of Gln to 2-OG, generating two molecules of glutamate (Glu), one of which may be used to produce other amino acids through the action of transaminases (Forde and Lea, 2007). Recently, in *A. thaliana*, Gln biosynthesis *via* GS1 was found to be related to increased tolerance to abiotic stress (Ji et al., 2019). Net NH_4_^+^ assimilation requires a source of 2-OG, which is generated through partial respiration of sugars. The provision of carbon skeletons for GOGAT functioning could be supplied when the citrate is exported from the mitochondria and transformed to isocitrate and then into 2-OG by the cytosolic NADP-ICDH enzyme. A number of observations corroborate the idea that cytosolic NADP-ICDH is the major isoform in green leaves and plays an important role in N-assimilation (Foyer et al., 2011). Levels of 2-OG can reflect C/N status and may play a signaling role in the co-ordination of carbon and nitrogen metabolism (Hodges, 2002).

Previous work using *G. monostachia* detached leaves showed that NH_4_^+^ was more effective than nitrate to positively modulate the increase in CAM expression under water stress. The expressions of ALMT and PEPC genes were intensified, and a higher nocturnal malic acid accumulation was observed. On the other hand, NO_3_^−^ supplied at various concentrations had little effect on the transcript number of *ALMT* and *PEPC* genes (Pereira et al., 2018). In the present investigation, we observed that when urea was supplied as the sole N source to the tank of *G. monostachia* plants, the nocturnal acid malic concentration was around five times greater than that in the presence of NH_4_^+^. After urea hydrolysis in the cytoplasm, the availability in CO_2_ may increase its incorporation by *PEPC1*, while more oxalacetate acid (OAA) and malate (through the action of NAD-MDH) can be produced, increasing acidity at night. Alternatively, during the day the CO_2_ generated by urea hydrolysis could be assimilated by ribulose-1,5-biphosphate carboxylase/oxygenase (Rubisco) in chloroplasts. In contrast, NO_3_^−^ treatment had no effect on C_3_-CAM shift, curiously showing the higher RWC after 21days of water deficiency. Therefore, urea may have a positive effect on both C and N metabolism of *G. monostachia*, mainly if we consider a CAM-idling plant with almost no access to CO_2_ from the atmosphere. For some tank bromeliads, urea can contribute 30% of the total plant N (Romero et al., 2010; Gonçalves et al., 2016). In nature, this organic N source could be excreted by various animals (for instance, anurans) that visit the bromeliad’s tank searching for shelter or a place to breed, and the leaf basal part assumed the role of roots in N absorption and urea hydrolysis (Takahashi and Mercier, 2011).

## Conclusion

Considering our biochemical and molecular findings (Figure 6), we conclude that drought had a positive effect on the C_3_-CAM shift in the apical leaf part, increasing the expression of genes related to carboxylation metabolism (*PEPC1*, *PPCK*, and *NAD-MDH*), movement of malate through vacuolar membrane (*ALMT9*) and decarboxylation process (*PEPCK*). Furthermore, our investigation revealed that urea can positively modulate the CAM-idling photosynthesis, which is characterized by the closure of stomata during the day and night. Our results reinforced that, when *G. monostachia* stay in long periods of drought, urea stimulates the expression of *PEPC1* and *ALMT9*, while *Urease* transcripts increase under the same conditions. At the same time, urea hydrolysis provides NH_4_^+^, which can be assimilated into Gln since our results showed increased *GS1* gene expression under water deficiency. We suggest that the link between C and N metabolism in *G. monostachia* may occur through the supplying of 2-OG by isocitrate dehydrogenase since the number of *NADP*-*ICDH* transcripts was also higher under drought. Thus, we suggest that urea nutrition provided in nature most by the symbiotic interaction of bromeliads with anurans may bring advantages to bromeliad survival under severe water stress, providing NH_4_^+^, which ultimately can produce amino acids and proteins, while CO_2_ can maintain photosynthetic efficiency, even when most stomata remain closed for 24h.

## Data Availability Statement

The datasets presented in this study can be found in online repositories. The names of the repository/repositories and accession number(s) can be found at: https://www.ncbi.nlm.nih.gov/, Bioproject ID PRJNA532595.

## Author Contributions

AG conceived, designed, and performed the experiments, analyzed the data, and drafted the manuscript. HM conceived and designed the experiments, analyzed the data, and drafted the manuscript. All authors contributed to the article and approved the submitted version.

## Funding

This work was funded by the Fundação de Amparo à Pesquisa do Estado de São Paulo (FAPESP) n° 2016/09699-5 to AG, n° 2011/50637-0 and 2018/12667-3 to HM and by the Conselho Nacional de Desenvolvimento Científico e Tecnológico (CNPq) n° 309504/2014-7 and 303497/2018-1 to HM.

## Conflict of Interest

The authors declare that the research was conducted in the absence of any commercial or financial relationships that could be construed as a potential conflict of interest.

## Publisher’s Note

All claims expressed in this article are solely those of the authors and do not necessarily represent those of their affiliated organizations, or those of the publisher, the editors and the reviewers. Any product that may be evaluated in this article, or claim that may be made by its manufacturer, is not guaranteed or endorsed by the publisher.

## Footnotes

^1^ https://www.blast2go.com/

^2^ https://software.broadinstitute.org/morpheus

^3^ http://perlprimer.sourceforge.net/
