# Supplementary material for: Transcriptomic and Biochemical Analysis Reveal Integrative Pathways Between Carbon and Nitrogen Metabolism in Guzmania monostachia (Bromeliaceae) Under Drought
Source: Front Plant Sci. 2021 Oct 8;12:715289. doi: 10.3389/fpls.2021.715289 (PMC8531410; doi:10.3389/fpls.2021.715289)
Supplement: Supplementary file 1 [file Table_1.doc]

**Supplementary Information**

**Table S1.** Analysis of variance (ANOVA) of the distinct effects of nutritional treatments and water treatments on the relative water content (RWC), on the phosphoenolpyruvate carboxylase (PEPC) enzyme activity, on the nocturnal acid accumulation (ΔH^+^), and on the gene expression of the CAM-specific gene (*PEPC1*) and the tonoplast aluminum-activated malate transporter gene (*ALMT9*) in the apical leaf portion of *Guzmania monostachia*. Nutritional treatments: nutrient solution without nitrogen source (control), Ca(NO_3_)_2_ (nitrate), (NH_4_)_2_SO_4_ (ammonium), and urea (urea)]. Twenty-four hours after the last application of the nutrient solutions, these bromeliads received two new water treatments for 21 days: distilled water (water) and water deficit condition in which the bromeliads did not receive distilled water (water deficit)

| Source of variation | d.f. | MS | *F* | P |
| --- | --- | --- | --- | --- |
| **RWC** |  |  |  |  |
| Nutritional treatments | 3 | 490 | 70.78 | **<0.001** |
| Water treatments | 1 | 5118 | 739.49 | **<0.001** |
| Nutritional treatments *vs.* water treatments | 3 | 20 | 2.95 | **0.053** |
| Residuals | 24 | 7 |  |  |
| **PEPC** |  |  |  |  |
| Nutritional treatments | 3 | 0.93 | 39.00 | **<0.001** |
| Water treatments | 1 | 6.90 | 287.24 | **<0.001** |
| Nutritional treatments *vs.* water treatments | 3 | 0.52 | 21.75 | **<0.001** |
| Residuals | 24 | 0.02 |  |  |
| **ΔH^+^** |  |  |  |  |
| Nutritional treatments | 3 | 21737 | 28.02 | **<0.001** |
| Water treatments | 1 | 48811 | 62.91 | **<0.001** |
| Nutritional treatments *vs.* water treatments | 3 | 20190 | 26.02 | **<0.001** |
| Residuals | 24 | 776 |  |  |
| ***PEPC1*** |  |  |  |  |
| Nutritional treatments | 3 | 1.42 | 82.48 | **<0.001** |
| Water treatments | 1 | 10.35 | 599.62 | **<0.001** |
| Nutritional treatments *vs.* water treatments | 3 | 1.19 | 69.14 | **<0.001** |
| Residuals | 24 |  |  |  |
| ***ALMT9*** |  |  |  |  |
| Nutritional treatments | 3 | 1.68 | 174.9 | **<0.001** |
| Water treatments | 1 | 12.26 | 1272.2 | **<0.001** |
| Nutritional treatments *vs.* water treatments | 3 | 1.23 | 128.6 | **<0.001** |
| Residuals | 24 | 0.01 |  |  |
