# Supplementary material for: Transcriptomic and Biochemical Analysis Reveal Integrative Pathways Between Carbon and Nitrogen Metabolism in Guzmania monostachia (Bromeliaceae) Under Drought
Source: Front Plant Sci. 2021 Oct 8;12:715289. doi: 10.3389/fpls.2021.715289 (PMC8531410; doi:10.3389/fpls.2021.715289)
Supplement: Supplementary file 2 [file Table_2.doc]

**Supplementary Information**

**Table S2.** Genes and primers of the **CAM-specific phosphoenolpyruvate carboxylase enzyme (*PEPC1*) and the** tonoplast aluminum-activated malate transporter **(*ALMT9*) of the** bromeliad *Guzmania monostachia* based on the sequences obtained from G. monostachia transcriptome (Mercier et al., 2019). Reference genes (*BAM*, *HSDD*) were selected since they were ultra-stable genes in the transcriptome (coefficient of variance < 0.07)

| **Functional category** | **Contig ID** | **Annotation** | ***Primers (5’ – 3’)*** | ***Amplicon* length (pb)** | **Efficiency (*slope*)** | **R^2^** |
| --- | --- | --- | --- | --- | --- | --- |
| ***PEPC1*** | c22549_g1_i2 | Phosphoenolpyruvate_carboxylase_1 | Forward: GAACGTCTTTGCTTTAGGACAC | 200 | 1.770 | 0.999 |
|  |  |  | Reverse: CCTGCCATATTCTAGTTCAGGTG |  |  |  |
| ***ALMT9*** | c31867_g1_i1 | Tonoplast aluminum-activated malate transporter_9 | Forward: GCAAAGGATTTAATCGGGGT | 171 | 1.747 | 0.999 |
|  |  |  | Reverse: ATAAGGCTTCATCGTCGGGTA |  |  |  |
| ***BAM*** | c14682_g1_i1 | Beta-amylase_2 | Forward: CTTTCTGGATGCGAAGGGTATC | 207 | 1.866 | 0.992 |
|  |  |  | Reverse: TGAAGGAAGGAGGAACACG |  |  |  |
| ***HSDD*** | c18630_g1_i5 | Beta-hydroxysteroid-dehydrogenase_decarboxylase_ 2 | Forward: CCTCAAGTCTCACTAACGAAG | 185 | 1.885 | 0.999 |
|  |  |  | Reverse: GATGTAGCAGCCGAATCAAC |  |  |  |
